# How Do Greeks Feel about Eating Insects? A Study of Consumer Perceptions and Preferences

**DOI:** 10.3390/foods13193199

**Published:** 2024-10-08

**Authors:** Alkmini-Anna Gkinali, Anthia Matsakidou, Anastasios Michailidis, Adamantini Paraskevopoulou

**Affiliations:** 1 Laboratory of Food Chemistry and Technology, School of Chemistry, Aristotle University of Thessaloniki, 54124 Thessaloniki, Greece; gkinalia@chem.auth.gr (A.-A.G.); adparask@chem.auth.gr (A.P.); 2 School of Agriculture, Department of Agricultural Economics, Aristotle University of Thessaloniki, 54124 Thessaloniki, Greece; tassosm@auth.gr

**Keywords:** adoption theory, insect-based foods, multivariate statistics, willingness to consume

## Abstract

Edible insects are considered among the most promising sustainable sources of protein to address the predicted deficiency of conventional food protein. Due to their nutritional and environmental benefits, there is an increasing interest in the ways insects could become part of the Western diet. Little is known about Greek consumers’ attitudes toward the habit of consuming insects as food. This study provides insight into Greek consumers’ preferences for insect-based food products. The data were collected through an online questionnaire (*n* = 1531). A two-step cluster analysis and a categorical regression were employed to classify the respondents into discernible clusters and determine the relationship between their socioeconomic characteristics and their willingness to adopt insect-based food products. Feelings of disgust and rejection were the predominant reactions to the concept of insects as food. The acceptance of novel foods derived from edible insects could be potentially enhanced by providing information regarding their positive effects, using familiar food products, and decreasing the insect’s degree of visibility by employing processed forms. Finally, the categories of insect protein-enriched food products (bakery, meat, snacks) that Greek consumers are more likely to consume were revealed. Such findings may be useful for promoting strategies regarding consuming insect-based products.

## 1. Introduction

With the world population expected to exceed nine billion by 2050, the demand for meat and meat products is projected to double. Feeding this huge population is going to be challenging, and the prospect of food insecurity has prompted researchers, entrepreneurs, and policymakers to discuss the broader exploitation of insects as food [1]. Insect consumption as well as the substitution of protein derived from conventionally farmed animals with insect protein offers numerous advantages. Insects exhibit higher feed-conversion efficiency, have lower requirements for land and water during the rearing process, and contribute less to greenhouse gas emissions, which makes them an environmentally friendly food source. Moreover, their nutritional value has been long recognized [2], and several articles have been published highlighting the nutrient composition of many edible insect species (i.e., high levels of polyunsaturated fatty acids, high-quality proteins, minerals, and vitamins) [3,4].

Even though entomophagy, the practice of eating insects, is widespread in approximately 80 countries across the world and ~2100 insect species are consumed daily by various ethnic groups, the acceptance of insects in Western countries is still quite low; thus, limited insect-based foods are consumed [1]. However, a growing interest in insects as food has been reported in Western countries in recent years, with many consumer studies published to examine the specific circumstances and conditions under which insects might be accepted by people in Western communities. Current literature highlights that food neophobia is the main factor that determines the unwillingness of European and North American consumers to accept insects as food. Disgust, fear, and rejection are the main reactions to the idea of insects as food [5,6]. Food neophobia is defined as the unwillingness to eat new foods, and it is an individual trait independent of the consumer’s culture. According to Barrena and Sánchez [7], the three main reasons related to the unwillingness to eat new foods are the feelings of aversion, danger, and disgust. It has been found that neophobia plays a pivotal and more significant role in shaping consumers’ decisions regarding the rejection of certain foods than other factors [8]. However, positive information about the taste of new foods increases the willingness to try them. Apart from taste, another determining factor is the familiarity of the consumer with the new food [7]. In Western countries, consumers perceive insects as dirty, primitive, and substandard sources of food [9] and therefore not suitable for the human diet [10,11]. Moreover, consumers are concerned about the safety and the presence of potentially harmful ingredients in insects, such as allergens, metals, and pesticides [10]. Nevertheless, it has been reported that consumers’ willingness to try a new food may be influenced by taste, nature, and food familiarity [7]. Other aspects of potential acceptability of insects as daily food that need to be taken into consideration, especially in countries that have strong culinary history and culture, such as the Mediterranean region countries, etc. [12], is what food items and recipes could be suitable for insect inclusion in order to be acceptable and finally be adopted. After all, there are indications that traditional ethnic culinary culture may be a factor in food neophobia [13].

Except for concerns related to food neophobia and safety, restrictive legislation remains one of the main obstacles to the acceptance of insects as food for humans in many Western countries [14]. All insect-based products fall under the category of ‘Novel Food’. Therefore, according to EU regulation 2015/2283 [15], a specific application to the European Commission, followed by EFSA’s scientific evaluation, is needed before the product is put on the market. Currently, the use of eight insect species is authorized: the black soldier fly (*Hermetia illucens*), the common housefly (*Musca domestica*), the yellow mealworm (*Tenebrio molitor*), the lesser mealworm (*Alphitobius diaperinus*), the house cricket (*Acheta domesticus*), the banded cricket (*Gryllodes sigillatus*), the field cricket (*Gryllus assimilis*), and the silkworm (*Bombyx mori*). These species are permitted as feed for aquaculture, poultry, and swine animals. Additionally, four insect species have been authorized for human consumption, namely ‘dried *Tenebrio molitor* larva’, ‘frozen, dried and powder forms of *Tenebrio molitor* larva’, ‘frozen, dried and powder forms of *Locusta migratoria*’, and ‘frozen, dried and powder forms of *Acheta domesticus*’ and ‘partially defatted powder’ of whole *Acheta domesticus* (house cricket) [16].

The most promising strategy for increasing the currently low acceptance of insects as food is to decrease the visibility of the insect by using processed insect [9]. In this way, the ‘yuck’ factor, which refers mainly to the Western attitude of overall disgust, fear, and repulsion to the idea of eating insects [17], is replaced by the familiar-looking [18]. For example, the meat as a rule is disguised in such a way that the animal is not distinct [18]. The findings of Lammers et al. [5] further support the idea that ‘hidden is best’ by showing that consumers are more willing to adopt non-visible insects in their daily diet in familiar-looking foods than to accept visible insects as food [19]. Puteri, Jahnke, and Zander [20] suggest that consumers could adopt insect-based products under the condition that the product’s ingredients are appropriate for human consumption. It has been proved that familiarity weakens neophobic reactions [21]. Another approach to diminish aversion towards entomophagy is educating consumers on its nutritional and environmental benefits [22]. People who received information about the various aspects of entomophagy tended to be less reluctant towards it after the seminar [21,23,24,25].

According to the survey conducted by Verbeke [26] in Belgium, 19.3% of 368 participants were willing to try edible insects (not further specified whether they were whole insects, visibly or invisibly processed) as potential meat substitutes in the future. The results also showed that the most likely early adopters were young adult males who were positive about trying new foods, interested in the environmental impact of their food choices, and only slightly attached to meat. Similarly, Schösler, de Boer, and Boersema [27] investigated the readiness of consumers in the Netherlands to adopt different types of meat substitutes, such as dishes with fried mealworms or locusts and pizza containing insect protein. In terms of attractiveness and likelihood of preparing them, the meat substitutes with visible insects were negatively rated compared to other choices. The pizza was rated more positively, especially by younger participants [27]. A survey conducted in Italy confirms that the primary reason for refusing to try edible insects is the disgust factor [24]. Also, the results of this study indicate that a tasting experiment had a positive influence on willingness to taste edible insects in the future. Moreover, it is suggested that a high level of education is a strong factor in improving the likelihood of trying insect-based products [28]. This could be explained by the stronger environmental awareness that highly educated people usually have compared to the less educated. Some authors investigated the level of knowledge about the sustainability of edible insects in a group of people originating from 14 countries, including Greece [29]. According to their results, higher knowledge is observed among males and young adults; in participants who reside in urban areas and countries such as Spain, Mexico, and Poland; and in participants with higher education levels and higher incomes. On the other hand, Greece was among the countries with the lowest knowledge about the environmental benefits of edible insects [29]. Although studies have been conducted about consumer acceptance of insects in many countries such as Italy, Belgium, and the Netherlands, the data are scarce concerning consumers’ reactions to insects as food in Greece.

When introducing innovative food products to a population, emerging new ideas or products spread across time following the diffusion process, as outlined in Rogers’ diffusion theory [30]. Rogers [30] proposes that five main elements influence the spread of a new idea: a. the innovation itself, b. adopters, c. communication channels, d. time, and e. the social system. According to Rogers’ theory, consumers can be categorized into five groups according to their attitude towards innovation: innovators (2.5%), early adopters (13.5%), early majority (34%), late majority (34%), and laggards (16%). The criterion for adopter categorization is innovativeness. This is defined as the degree to which an individual adopts a new idea relatively earlier than other members of a social system [31]. The group classified as ‘Innovators’ are eager to try new ideas, and their interest in new ideas leads them out of a local circle of peers. The ‘Early adopters’ tend to be integrated into the social system more than ‘Innovators’, and usually they help to accelerate the diffusion of the new idea. The people belonging to the ‘Early majority’ category needs more time to adopt a new idea and will adopt it just before the average member of a social system. Finally, the group of people in the ‘Late majority’ are skeptical, while the ‘Laggards’ are traditionalists and the last to adopt an innovation.

Agriculture and food production in Greece has demonstrated large growth in the last decade, and the sector is characterized as a ‘rising star’ for the Greek economy. The city of Thessaloniki, where AUTH, the largest university in the country, is located, is the second largest city in Greece and is the capital of the prefecture of Kentriki Macedonia. The region exhibits the second largest entrepreneurship activity in Greece. Moreover, it is one of the most important agricultural production centers in EU, demonstrating a high production of both animal and vegetable products, along with a high food manufacturing activity [32]. Therefore, the region combines a high level of entrepreneurship with a major agricultural and food production background, thus indicating the great importance of understanding consumers’ attitudes in this area for introducing a novel food. The study focused on collecting a representative sample from all 13 municipalities of Thessaloniki to include both urban and rural areas of the region.

The aim of this study was to collect data concerning the profile and characteristics of consumers living in Thessaloniki, Kentriki Makedonia, where entrepreneurship and agrifood sector are important parts of the local and national economy, and understand the framework under which they would consider insects as food. More specifically, the objectives of the conducted survey were:

- to examine the willingness of Greek consumers residing in Thessaloniki to incorporate insects into their diet,
- to identify the factors (e.g., socio-demographic data, food neophobia features, sustainability consciousness, etc.) that may shape consumers’ attitudes towards entomophagy,
- to confirm the significance of disguising insects before incorporating them into food to enhance acceptance of insect-based foods
- to investigate whether providing information regarding the benefits of consuming edible insects could influence willingness to adopt them as food, and
- to explore the insect-based food items that are more appealing to consumers to better comprehend the gastronomical aspects of their preferences.

To gain deeper insight into consumer profiles, the study employed the Rogers diffusion theory [30], as edible insects are considered novel food products.

## 2. Materials and Methods

In this study, an online questionnaire, due to COVID-19 restrictions, was used to collect survey data concerning the willingness of randomly selected consumers. The online questions were designed in a compliant manner (fully structured with predefined answers), and the study protocol was previously approved by the Ethics Committee of the Aristotle University of Thessaloniki (#224298/2020). The notification call of the survey to fill in an online questionnaire was sent via the university administration e-mail network (>5000 recipients) and communicated via authors’ personal social networks (Facebook and LinkedIn pages), from November to December 2020. The participants should be residents of the region of Thessaloniki (13 municipalities with a total population >1 × 10^6^ [33] and >18 years old. No reminders were sent. All respondents participated voluntarily without any monetary or other kind of compensation for their involvement. Fourteen participants were excluded due to response inconsistency or non-logical answers. Data will be made available on reasonable request. Finally, a representative sample of 1531 questionnaires were included in the final dataset [34] with a 5% error estimation.

The structure and content of the questionnaire was based on prior studies focused on insect consumption and consumer perception. Apart from the demographic and socio-economic status of the participant-related data [5,18,24,28,35,36,37,38,39], their familiarity with the practice of eating insects as food [5,18,25,37], and their willingness to practice it [24,35,37,38,39]. Researchers, additionally, explored the reasons behind participants’ attitudes [5,24,25,28,35,38,39] and investigated the types of insect-based food products that consumers would be more willing to accept [5,37]. Furthermore, as indicated by several studies [24,25,36,40], consumers’ attitudes towards eating insects can be affected through the provision of positive information. In the present study, the participants did not receive any prior information about the subject of the survey, but brief information regarding the advantages of eating insects was provided before the final section of the questionnaire.

In particular, the questionnaire was divided into four sections:

Section A: General questions:

- gender, age, education, marital status, occupation, income, and several other personal, socioeconomic, and demographic characteristics.

Section B: Questions about entomophagy:

- familiarity with the practice of entomophagy and willingness to learn about it,
- willingness to adopt visible or/and non-visible insect-based products,
- reasons regarding the adoption and non-adoption of visible or/and non-visible insect-based products.

Section C: Brief education and training, including information and facts about:

- nutritional advantages,
- environmental benefits (lower greenhouse gas emissions and less land usage compared with conventional livestock like beef, pork, and, poultry),
- social aspects of entomophagy within ethnic groups around the world,
- safety issues.

Section D: Questions about entomophagy following the brief education and training (Table S1, Supplementary Materials):

- willingness to adopt products based on visible and/or non-visible insects after providing information and
- willingness to consume specific food products containing visible and/or non-visible insects.
- The collected dataset was quantitatively analyzed using summary statistics and multivariate statistical analyses.

The selected independent variables applied for data collection and analysis are presented in Table 1.

The investigation of the drivers that influence consumers’ decision to adopt insect-based products is related to pre-defined elements, achieved by employing both descriptive statistics and multivariate statistical analysis. In particular, a two-step cluster analysis (TSCA) was used to classify the respondents into distinct clusters in order to explore the different levels of willingness to adopt insect-based products (whether containing visible or/and non-visible insects). Specifically, the TSCA automatically classified the respondents using their responses to the following questions: (a) knowledge about the practice of entomophagy, (b) willingness to be educated about entomophagy, (c) willingness to adopt visible insect-based products, (d) willingness to adopt non-visible insect-based products, (e) willingness to adopt visible insect-based products after the brief education/training of Section C of the questionnaire, and (f) willingness to adopt non-visible insect-based products after the brief education/training of Section C of the questionnaire. The pre-defined answers for questions about ‘knowledge of the practice of entomophagy’ and ‘willingness to be informed about entomophagy’ were: ‘yes’, ‘not sure’, and ‘no’, where participants who responded with ‘yes’ were considered to have knowledge about the practice of entomophagy and to be willing to be informed about it. The participants answered several questions regarding their dietary, environmental, and daily practices using a five-point scale (1 = very often, 5 = never). Descriptive statistics were employed to assess the mean scores for each cluster for statements concerning dietary, environmental, and daily habits were estimated. To better comprehend consumer behavior and effectively predict their attitudes towards insect-based food products, the participants answered several questions regarding the reasons for the adoption and non-adoption of visible or/and non-visible insect-based products using a five-point scale (1 = very often, 5 = never). Descriptive statistics were employed to assess the mean scores for each reason for adoption and non-adoption of visible or/and non-visible insect-based products.

Additionally, categorical regression [41] was applied to effectively handle optimally transformed categorical variables to determine the relationship between consumers’ characteristics and their willingness to adopt insect-based products.

Finally, the respondents were also asked to report their willingness to consume specific insect-based products containing edible insects in a visible or a non-visible form, using a five-point scale (1 = strongly disagree, 5: strongly agree). Descriptive statistics were employed to assess the mean scores for each insect-based product. IBM SPSS Statistics 27.0 was used for data analysis.

## 3. Results

The majority of the participants who responded to the research team’s call and filled out the questionnaire were females (>60%) (Table 2). It has been reported that females, in general, tend to be more sensitive to environmental, animal-welfare, and nutritional (especially meat consumption-related) issues [42,43]. This attitude may be a reason for the higher interest of women in participating voluntarily in the survey compared to males.

### 3.1. Cluster Segmentation and the Role of Brief Education and Training

Cluster analysis was employed to reveal natural groupings of consumers within the given set of respondents. The Two-Step Cluster Analysis (TSCA) automatically classified the respondents into 5 different clusters, according to the Bayesian Information (BAIC) Criterion [44]. The segmentation in relation to Rogers’ theory was applied depending on their responses. For example, cluster 1 was classified as ‘innovators’, because it demonstrated a high level of willingness to learn about the practice of entomophagy (92.1%) and to consume insects in both visible and non-visible forms, before and after brief education. In contrast, the group that showed little interest in learning about entomophagy, with only 5.7% willing to learn, and unwillingness to consume insects was classified as ‘laggards’. Thus, the obtained clusters were categorized into five general groups: ‘innovators’, ‘early adopters’, ‘early majority’, ‘later majority’, and ‘laggards’, according to their response patterns as suggested by Rogers [30] (Table 2). This categorization relies on the adoption/diffusion of of” innovation theory” in order to comprehend the process that occurs as consumers adopt novel products. Thus, the majority of the respondents (32.1%) were classified within the third cluster (‘early majority’), 18.9% and 19.4% were included in the first (‘innovators’) and fifth (‘laggards’) clusters, and 14.3% and 15.3% were assigned to the second (‘early adopters’) and fourth (‘late majority’) clusters, respectively, depending on their responses (Table 2).

Familiarity with the practice of entomophagy has been indicated as a significant predictor of the willingness to adopt insects as food [21,26]. Participants who claimed familiarity were 2.6 times more likely to adopt insects than those who reported they had never heard about it [26]. Table 2 indicates that the majority of the participants in each cluster had heard the practice of consuming insects as food before the survey, but they did not know its exact meaning (47.9–53.0%). However, it is noteworthy that most of the participants (38.7%) in the ‘laggards’ cluster had no knowledge about the practice of entomophagy. As expected, ‘innovators’ and ‘early adopters’ were more willing to learn about entomophagy (92.1% and 96.8%, respectively). The willingness to be educated regarding entomophagy is the key factor that differentiated the ‘late majority’ from the ‘laggards’. Among the ‘late majority’, a group representing 62.8% was willing to get informed, while within the ‘laggards’, only 5.7% were willing to get informed, even though both groups were negative in adopting entomophagy.

Regarding the level of willingness to adopt either visible or non-visible insect-based products, 32.8% of ‘innovators’ (290 participants) were willing to adopt visible insects, while 100% were willing to adopt processed insects. However, after a brief education about the benefits of entomophagy, 49% of them responded positively to the idea of consuming food products with visible insects. In the case of ‘early adopters’ (219 participants), the vast majority responded ‘maybe’ to both questions regarding visible (91.3%) and non-visible (93.6%) insects. After the brief education about entomophagy advantages, 33.3% and 52.1% had a positive response about consuming visible and non-visible insect-based products, respectively. Regarding the ‘early majority’ participants (491), 94.9% had a negative response to visible insects, and 70.9% had an intermediate response (‘maybe’) to non-visible insects. Also, providing information about the benefits of the entomophagy had a positive effect. More specifically, 31% responded with ‘maybe’ and 8.4% responded positively about visible insects, while 41.5% had a positive response about the adoption of non-visible insect-based products. On the contrary, both the ‘late majority’ (234) and the ‘laggards’ (297) denied consuming insects (~100%), regardless the insect form and whether they were educated about the benefits of entomophagy.

In general, the impact the brief education affected the clusters differently. Present results revealed that even a brief education highlighting the positive features of entomophagy increased the willingness of the ‘innovators’, ‘early adopters’ and ‘early majority’ to consume insect-based products in the future. More specifically, as demonstrated in Table 2, 32.8% of the ‘innovators’ cluster responded ‘yes’ to the statement ‘I would consume food products that contain insects in a visible form in the future’. After the brief education, 49.0% of the ‘innovators’ cluster responded ‘yes’ in the same statement. As far as the ‘early adopters’ cluster, the percentage of the participants who responded ‘yes’ to the statement ‘I would consume food products that contain processed insects in an invisible form in the future’ increased remarkably from 1.4 to 33.3%. Finally, in the case of the ‘early majority’ cluster, the percentage of the participants who responded ‘yes’ to the statement ‘I would consume food products that contain processed insects in an invisible form in the future’ increased dramatically from 7.1 to 41.5%. Therefore, in the participants who were initially reluctant to adopt insect-based products, the brief education regarding entomophagy weakened their hesitance and reversed their initial attitudes against it, as they became aware of the benefits and were reassured about their safety concerns. The present study confirmed previous findings [6,24,25,36,45,46], which indicated that providing information about the benefits of edible insects can enhance their acceptance. However, in the present study, it is indicated that this strategy is mostly effective towards the ‘innovators’ and ‘early adopters’ clusters, while the negative opinion of the ‘late majority’ and ‘laggards’ clusters remained practically unchanged following the brief educational intervention.

On the other hand, as expected, the majority of the participants were more willing to adopt insect-based products incorporating edible insects in powder form that would make them invisible. These findings are in line with previous reports, which support that, in Western countries, insects are more easily accepted when they cannot be visually identified by consumers [5,11,45].

### 3.2. Cluster Comparison

#### 3.2.1. Socio-Demographic Characteristics

Table 3 shows the participant percentages within each cluster that correspond to the listed responses. The results show that, generally, the clusters are composed mainly of female participants (53.4–70.5%), who lived in Thessaloniki (50.5–57.2%). Their ages ranged from 26 to 45 (48.7–59.4%), they had no children (51.2–69.3%), held a college/university degree, and had a monthly income in the range of EUR 1501–2200. However, the ‘innovators’ cluster exhibits a higher percentage of male participants (45.2%) that are younger compared with the other cluster (30% of them are between 18 to 25). Nearly 70% of them do not have children, and over one-quarter (25.9%) reside in one-person households, with 32.8% being university students. The ‘early adopters’ mainly consist of female participants (61.6%) with a higher educational level, as 78.1% of them have graduated from college/university. On the contrary, the ‘laggards’ have the highest percentage of participants aged over 46 (36.4%). Moreover, over half of the ‘late majority’ (53.4%) and ‘laggards’ (51.2%) have at least one child, with their households composed of 3 (25.2%) and 4 (28.6%) members, respectively, while only 17.9 and 16.5% of them, respectively, were college/university students.

Many studies have already shown that gender may influence the willingness to eat insect-based products [26,28,47]. Males and younger participants were more likely to adopt insects as a meat substitute [26]. Also, males displayed a higher willingness to consume visible insects than women [5] and could potentially be more open to considering insects as alternatives to conventional protein sources [24]. A possible explanation for the observed gender difference may be that females tend to be more afraid of and disgusted by insects [5,26]. Aside from gender, age has been also reported to be an important factor affecting the willingness to consume insects. Youngsters appear to hold a more positive outlook on the idea of entomophagy [26,35]. For example, young people in the Czech Republic were more willing to taste samples of edible insects [48]. That could be possibly explained by the fact that young consumers are more concerned about the environmental consequences of their dietary choices and are more open to experiencing novel foods [10].

No meaningful differences in education and monthly income were observed between the clusters. The majority of individuals across the groups were college/university graduates (67.2–78.1%). Notably, the ‘early adopters’ appeared to be composed of slightly more graduates (78.1%) compared to the other groups. According to Cicatiello et al. [28], consumers holding a university degree had about 8 times higher probability of agreeing to try insects than those with a lower education, but Zhou et al., argued the educational level significantly influenced the willingness to consume insects [5].

Regarding employment status, the ‘innovators’ group had the highest percentage of university students (32.8%), while the group of ‘early adopters’ demonstrated the highest percentage of unemployment (12.8%) compared to the other groups.

#### 3.2.2. Dietary, Environmental, Daily Habits, and Allergies

Dietary and environmental consciousness [26] are important factors in identifying the potential adopters of edible insects. Table 4 shows the mean scores of each cluster for statements concerning allergies. Although food allergies have been reported to affect food-related behaviors [49], the present study revealed similar percentages of individuals belonging to ‘innovators’ and ‘laggards’ groups who reported having fish/seafood allergies (2.8% and 2.7%, respectively). This indicates that fish/seafood allergies may not significantly affect the willingness to eat insects. Conversely, people with insect allergies might be more skeptical about consuming insects as food, as was revealed by the present study, where similar percentages of individuals belonging to the ‘innovators’ and ‘laggards’ groups reported having fish/seafood allergies (2.8% and 2.7%, respectively).

Table 5 shows the mean scores of each cluster for statements concerning dietary, environmental, and daily habits. ‘Innovators’ frequently consumed meat products (mean 2.49), consumed dairy products (mean 2.09), recycled food waste (mean 2.00), and read food product labels (mean 2.25). ‘Early adopters’ consumed slightly fewer meat products (mean 2.68), but they consumed organic products more often (mean 3.21). ‘Laggards’ appeared to consume meat products less frequently (mean 2.76) compared to the other clusters. The ‘early majority’, ‘later majority’ and ‘laggards’ exercise even less frequently (mean 3.22–3.29) and do not recycle food waste so often (mean 2.14–2.20). All clusters show a moderate consuming frequency of fish products (mean 3.13–3.29), with ‘innovators’ consuming them more frequently.

According to Rovai et al. [45], a varied diet, without dietary restrictions, indicates a higher acceptance of insects as food. Moreover, an interesting finding is that the consumption of fish products, especially raw fish/sushi, is associated with a higher possibility of accepting edible insects as a food source [45]. Additionally, the tendency of ‘laggards’ to decrease meat product consumption could possibly justify their lower acceptance of insects. Some authors suggest that the intention to reduce meat consumption is a significant predictor of the willingness to consume insects [18,26], while others suggest that meat consumption is not linked to the willingness to consume insect-based food products (Lammers et al., 2019) [5]. The frequency of consuming organic products was not identified as a significant factor in the willingness to consume insects [28].

Regarding the environmental consciousness of the consumers, it has been found that the environmental sustainability and impact of the foods they consume are not driving factors associated with their willingness to eat insects [5,45,50]. However, a recent study by Hartmann, Ruby, Schmidt, and Siegrist [51] shows that consumers of insect-based food products are perceived as more environmentally and health-conscious than meat consumers.

### 3.3. Categorical Regression Analysis Model

To uncover survey participants’ choices regarding the adoption of visible or/and non-visible edible insects as food, and to determine how these decisions are influenced by their personal characteristics, two categorical regression (CATREG) models were run (Table 6). The categorical regression models yielded R values of 0.417 and 0.405, respectively, indicating a moderate relationship between the ‘tendency for potential adoption of insect-based products’ and selected predictors. However, since R2 is equal to 0.174 and 0.164, respectively, it is evident that 17.4% and 16.4% of the variance in the ranking of the ‘tendency for potential adoption of insect-based products’ can be explained by the regression of the optimally transformed variables used. The F statistic values of 6.23 and 10.50, with corresponding a = 0.00, indicate that these models are performing well.

The relative importance measures [52] of the independent variables indicate that the most influential factors predicting the dependent variables for model Ι are ‘occupation’ (accounting for 34.7%), ‘monthly income’ (14.7%), ‘number of children’ (14.7%), ‘municipality of residence’ (12.1%), ‘age’ (11.3%), and ‘number of household members’ (9.7%). The cumulative importance of these six variables accounts for about 97%. Similarly, for model ΙΙ, the most influential factors predicting the dependent variables are ‘consumption frequency of seafood products’ (41.9%), ‘checking food label frequency’ (20.7%), and ‘body exercising frequency’ (19.6%). The cumulative importance of these three variables is about 82%.

Finally, the tolerances of all variables are high enough to assure exclusion of the multicollinearity problem (Table 6).

**Figure 1:**
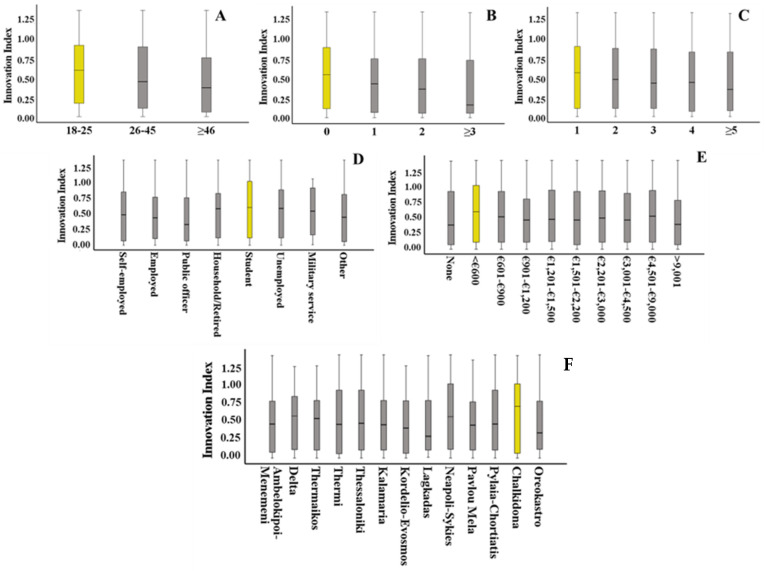
Socio-demographic characteristics of the participants, with high importance, according to regression model I (*R*^2^ = 0.174): (**A**) age, (**B**) number of children, (**C**) number of household members, (**D**) occupation, (**E**) monthly income, and (**F**) municipality of residence.

**Figure 2:**
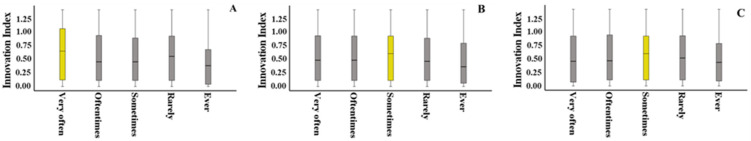
Diet and behavior habits of the participants, with high importance, according to regression model II (*R*^2^ = 0.164): (**A**) consumption of fish/seafood products, (**B**) body exercising, and (**C**) food label checking.

The CATREG model I analysis results are presented in Figure 1. Regarding the place of residence, the participants living in the municipality of Chalkidona seem to be more willing to adopt insect-based food products in the future. That attitude can be attributed to the fact that Chalkidona residents are mainly occupied in the agricultural sector. Additionally, regardless of their place of residence, respondents who are university students aged between 18 and 25, have no children, live in one-member households, and have a monthly income less than EUR 600 are more likely to adopt insect-based products in the future. The results of CATREG model II (Figure 2) showed that participants who consume fish and seafood products very often, exercise, and occasionally check food labels are more likely to adopt insect-based products in the future.

The role of socio-demographic factors in shaping consumer behavior towards insect-based food products is not clearly defined in the literature. In the present study, young age (18–25), not having children (number of children = 0), living alone (household member = 1), and having a low monthly income (<EUR 600) came out as strong factors that increase the likelihood to consider adopting insect-based food products in the future. In line with the fact that youngsters are keener on/eager to engage in entomophagy, it was justifiable to find that, as far as the ‘occupation’ factor, university students are more likely to accept insects as food. Also, the correlation between education and the willingness to consume insects may be explained by the stronger environmental awareness that young and highly educated people usually have [28]. In general, young people, such as university/college students, are more sensitive to the current challenges related to food sustainability and will more likely be receptive to the idea of adopting insect-based products [53].

### 3.4. Reasons for Adoption and Rejection of Insect-Based Products

Table 7 presents the rationales behind adopting insect-based food products. Such data would prove valuable in planning marketing strategies for these products. Notably, the most important reason for potentially adopting insect-based food products, regardless of insect visibility, is that they are considered an alternative protein source (mean value ~4.3). Curiosity drives many of the participants to consider insect consumption, particularly when the insects are in a visible form (mean = 4.14 for visible insects and mean = 3.95 for non-visible, *p* < 0.05)). Insects’ high nutritional value also motivates many potential consumers to consider consuming either visible or non-visible insects (mean = 3.99 and mean = 3.92, respectively). In addition, it seems that sensitivity towards animal welfare (means 3.91 and 3.80) as well as environmental considerations (means 3.72 and 3.92) may push consumers to choose insects as food, whether visible or non-visible. Insect visibility seemed to play an important role (*p* < 0.05) when replacement of other products with insects and nutrition habitude are considered as factors of adoption.

Generally, in people from Western countries where entomophagy is uncommon, such as Italy and Hungary, curiosity is the main driver pushing them to try insect-based food products [28,54]. According to Sogari et al. [24], Italians’ decision to taste a cookie made with cricket flour is mostly curiosity-driven concerning its taste and texture (mean 4.44 ± 0.66), while also being thought of as an alternative source of protein (mean 3.79 ± 1.01). Moreover, Gere et al. [18] found that Hungarian consumers who are seeking new food options and intend to reduce meat intake might be more receptive to insect-based products. These attitudes are in line with this study’s findings.

On the other hand, the main factors contributing to hesitation regarding entomophagy, regardless the form in which insect is present in the food (Table 7), were the feeling of disgust (mean = 4.90 and mean = 4.83, corresponding to visible and non-visible forms), fear (mean = 3.97 and mean = 4.03, corresponding to visible and non-visible forms), and the respondents’ unwillingness to change their nutritional habits (mean = 3.94 and mean = 3.93, corresponding to visible and non-visible forms). Notable, but not unexpected, was that appearance is the second most important barrier to accepting insects in a visible form as food (mean = 4.38). A recent study, conducted among Greek adults from the Generation Z cohort, revealed that the primary reason for rejecting the adoption of insect-based products is disgust [37]. As ‘food neophobia’ is defined as the unwillingness to eat new foods, according to Barrena and Sánchez [7], the three main reasons related to this reluctance are the feelings of aversion, danger, and disgust; the present study indicated, by the obtained high score of these responses, that food neophobia may play an important role in accepting insects as food by Greek consumers. Studies conducted in other countries also indicated that food neophobia and disgust reactions are particularly strong towards edible insects due to their aversive sensory properties, especially when served as whole bodies [6,11,24,25,26,45,55]. Moreover, a low acceptance rate is related to the perception of insects as a ‘primitive’ food source, typically eaten by people of low economic status [9]. Cicatiello et al. [28] identified appearance as the primary barrier to insect consumption by individuals from Italy Additionally, respondents in this study were concerned about safety issues (4.18 on average). However, consumers’ responses depend on the form in which the products are presented to them, and the likelihood of their acceptance generally increases with visibility degree [26].

### 3.5. Products

**Figure 3:**
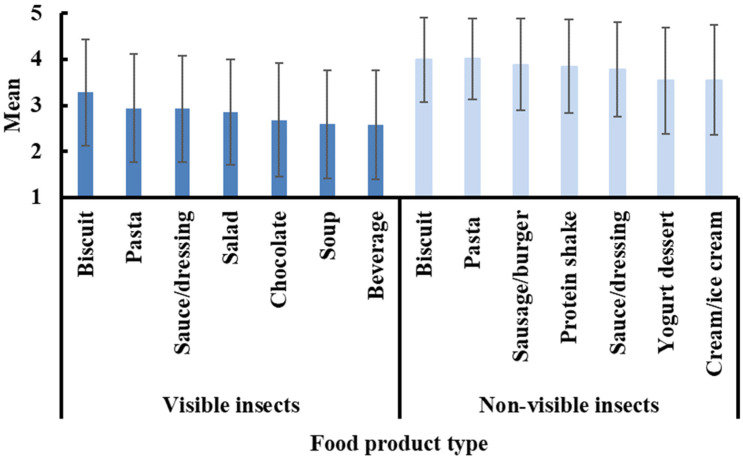
Participant preferences for insect-based products with visible and non-visible insects. Rating scale notes: 1: strongly disagree, 2: disagree, 3: neutral, 4: agree, 5: strongly agree.

The respondents were also asked to report their willingness to consume specific insect-based products, including (a) biscuits, (b) pasta, (c) sauces or dressings, (d) salads, (e) chocolates, (f) soups, and (g) beverages (alcohol or juice), all containing edible insects in a visible form. These products were selected to cover a range of foodstuff that are widely consumed in Greece and Europe, as well as (a) pasta, (b) biscuits, bread, cakes (bakery products), (c) sausages, burgers (meat analogs), (d) protein shakes, (e) sauces or dressings, (f) yogurt desserts, and (g) creams or ice creams containing edible insects in a non-visible form. The suggested insect-based products with non-visible insects were selected based on products already available in European markets, so they would be familiar to consumers. Also, there were products included that use protein as the basis of their formulae (i.e., salad dressings), regarding which previous studies have shown great potential of applications [56]. Figure 3 summarizes the acceptability scores of these insect-based products. As expected, respondents’ reactions were more positive in the case of food products containing insects in a non-visible form (3.54–4.01) compared to those with visible insects (2.57–3.28). More specifically, participants were more willing to consume biscuits (mean 3.28) and pasta (mean 2.94) containing visible insects. A similar trend was observed in the responses regarding pasta and bakery products with insects in a non-visible form, which collected the highest scores, 4.01 and 3.99, respectively, which was also observed by another. In addition, meat analogs, protein shakes, and sauce/dressing products received high mean values (3.89, 3.85, and 3.78, respectively).

Lombardi et al. [6] investigated consumer preferences for three food products, i.e., pasta, cookies, and chocolate bars with insects, and their findings are in line with the results of the present study. However, they rejected the idea of eating cookies and chocolate bars containing insects, which might indicate that the incorporation of edible insects in sweet preparations, such as in a dessert or in a chocolate bar, which is generally consumed for pleasure, would be considered inappropriate by consumers [57].

### 3.6. Strategies

Up to now, in Europe, the acceptance of edible insects has been low. Legal restrictions and classifying edible insects as novelty foods have resulted in a limited availability of edible insect-based food products in EU markets. Therefore, consumers remain unfamiliar with the idea of eating insects and thus reluctant, as confirmed by this study. To overcome consumers’ food neophobia and the feeling of disgust towards edible insects, successful strategies previously applied when introducing new food products to the market need to be adopted [21].

In general, familiarity plays a key role in shaping consumer preferences [51]. The acceptance of edible insects is likely to increase with increasing familiarity with the product [10,21] due to fewer food neophobic reactions [58]. Even basic information and reassurance about the benefits and safety of the habit of entomophagy, as revealed by the present study, could influence the attitudes of consumers towards the idea of eating insects (Table 2). Furthermore, the incorporation of insects in familiar products is expected to enhance their acceptance [55]. As such, the current market strategy aims to develop insect-based food products with non-visible insects that are common in Western diets, such as burgers, bread, biscuits, pasta, crackers, crisps, candy bars, shakes, soups, and sauces [19]. However, the insect-based products that Greek consumers reported that they were more willing to try were convenient food products such as biscuits and pasta, both with insects in visible and non-visible form (Figure 3). In Greece, consumers’ preferences could possibly be related to their traditional cuisine. House [59] suggests that insects should be introduced to Western markets using authentic, traditional cuisine and should not be incorporated into Western products. Therefore, the marketing strategy should follow an approach similar to the successful marketing strategy applied to sushi in the USA during the 1960s.

Based on participants’ responses, the main reasons for rejecting food products containing insects are disgust and fear (Table 7). Studies have shown that the attitude of disgust is learned at a young age and passed down through generations [46]. Moreover, educating school children about taste led to a reduction in food neophobia and increased their willingness to try novel foods [10]. To attract consumers who reject edible insects due to disgust, Bakalloglou [46] suggested including information about the nutritional and environmental benefits of edible insects in school education. Furthermore, providing information to parents will increase their children’s familiarity with edible insects and affect their future consumer approach [60].

According to Kamenidou et al. [37], the initial step in marketing insect-based food products in Greece is to make Greeks aware of entomophagy’s benefits, e.g., the nutritional value of insects, through communication campaigns. Apart from sharing information and increasing familiarity, a first positive taste experience is a determinant factor in the introduction of insects as a food source [5,9,10]. Thus, the Greek market needs to create opportunities for consumers to try insects as food in order to generate positive experiences. Tasting edible insects is necessary for boosting consumers’ familiarity with their taste and texture, aiding them to overcome their fear of insect consumption [10]. Also, an appealing appearance of the product, the packaging, and the advertisement can further help to reduce the inhibition threshold for first consumption [61].

Finally, marketing strategies should address the environmental and sustainability aspects and primarily focus on marketing messages concerning the health benefits of insects’ consumption [5]. Communication strategies are recommended to increase awareness and provide information about entomophagy and its advantages.

## 4. Conclusions

This study is exploratory and contributes to the existing literature on entomophagy in Greece; however, it does have some unavoidable limitations. The study focuses on the region of Thessaloniki, so extending the research to other areas of the country would be of interest. One limitation is the demographic distribution, specifically gender bias, as most of the participants are females. Moreover, all questions regarding acceptability and willingness to adopt insect-based products were conceptual and not validated with real food products or sensory evaluation. Despite these limitations, this study provides extensive insight into the behavior and attitudes of the Greek consumers, filling an information gap for marketers, and thus signifying future trends in the Greek entomophagy market.

Τhe relatively high percentage of ‘innovators’ in the present study indicates that the adoption of insect-based food products by Greek consumers is not in a primary phase. Therefore, ‘early adopters’ and the ‘early majority’ should receive more marketing attention, while the ‘innovators’ are ready to accept insect-based products. Nevertheless, two main axes of marketing strategies should be considered before introducing insect-based food products into the Greek market. Education and familiarization of the consumers with the concept of eating insects should play a pivotal role in insect-based food marketing. Additionally, it is conceivable that food products incorporating invisible insects would receive higher acceptance rate by Greek consumers and therefore advertising and product development should focus on these activities.

## Figures and Tables

**Table 1:** Selected independent variables.

Independent Variables	Type	Categories
Knowledge of the practice of entomophagy	String (nominal)	yes, not sure, no
Willingness to be informed about entomophagy	String (nominal)	yes, not sure, no
Classification of respondents	Ordinal	1—innovator, 2—early adopter, 3—early majority, 4—later majority, 5—laggards
Municipality of residence	String	Thessaloniki, Ambelokipi-Menemeni, Delta, Thermaikou, Thermi, Kalamaria, Kordelio-Evosmos, Lagadas, Neapoli-Sykies, Pavlou Mela, Pylea-Chortiati, Chalkidonos, Oreokastro
Area of origin	String	City, rural town, village
Age	String	18–25, 26–45, >46
Gender	String	male, female, no answer
Number of children	Numeric	0, 1, 2, >3
Number of household members	Numeric	1, 2, 3, 4, >5
Education	String	Illiterate, elementary school graduate, high school graduate, high school/6-grade high school graduate, graduate of postsecondary technical school, technical/vocational high school graduate, graduate of vocational education institute university/college student graduate of university/college
Occupation	String	Public officer, employee, self-employed, university/college student, household/retired, unemployed, military service, other
Monthly income	String	None, <EUR 600, EUR 601–EUR 900, EUR 901–EUR 1200, EUR 1201–EUR 1500, EUR 1501–EUR 2200, EUR 2201–EUR 3000, EUR 3001–EUR 4500, EUR 4501–EUR 9000, >EUR 9001

**Table 2:** Two-step clustering characteristics of the level of knowledge about entomophagy, willingness to be educated about it and willingness to consume insect-based products.

	Innovators	Early Adopters	Early Majority	Late Majority	Laggards
Cases, *n* = 1531	290	219	491	234	297
%	18.9	14.3	32.1	15.3	19.4
	Percentages %				
Level of knowledge about entomophagy					
Yes/Not sure	27.6/47.9	16.9/52.5	7.5/53.0	0/52.6	28.6/32.7
Level of willingness to be educated about entomophagy					
Yes/Maybe	92.1/5.5	96.8/0	56.6/23.2	62.8/37.2	5.7/2.0
Level of willingness to consume insects in a visible form					
Yes/Maybe	32.8/58.6	8.7/91.3	0.2/4.9	0/0.4	0/0.3
Level of willingness to consume insects in a non-visible form					
Yes/Maybe	100/0	1.4/93.6	7.1/70.9	0/0	0/0
Level of willingness to consume insects in a visible form after brief education					
Yes/Maybe	49.0/37.9	33.3/45.2	8.4/31.0	0/0	0.3/0
Level of willingness to consume insects in a non-visible form after brief education					
Yes/Maybe	92.4/6.2	52.1/37.9	41.5/46.8	0/0	0/0

**Table 3:** Socio-demographic characteristics (%) of the participants (*n* = 1531).

	Total	Innovators	Early Adopters	Early Majority	Later Majority	Laggards
Cases	1531	290	219	491	234	297
%	100	18.9	14.3	32.1	15.3	19.4
	Percentages %					
Municipality of residence						
Thessaloniki	53.9	57.2	53.0	54.8	53.0	50.5
Area of origin						
City	70.5	69.0	68.9	71.9	68.4	72.4
Rural town	12.2	12.4	15.5	12.8	11.1	9.4
Village	17.3	18.6	15.5	15.3	20.5	18.2
Age						
18–25	21.9	30.0	14.2	28.1	17.9	12.8
26–45	52.3	49.3	59.4	48.7	58.5	50.8
≥46	25.8	20.7	26.5	23.2	23.5	36.4
Gender						
Female	63.2	53.4	61.6	67.4	70.5	61.3
Male	36.0	45.2	36.5	32.0	29.1	38.7
Prefer not to answer	0.8	1.4	1.8	0.6	0.4	
Number of children						
0	61.6	69.3	62.1	67.0	53.4	51.2
1	12.7	10.0	13.2	11.6	16.7	13.5
2	20.4	16.9	19.6	17.3	23.5	26.9
>3	5.4	3.8	5.0	4.1	6.4	8.4
Number of household members						
1	20.8	25.9	21.9	18.3	18.8	20.9
2	25.0	23.4	24.2	29.1	22.2	22.6
3	19.1	16.9	20.5	18.3	25.2	16.8
4	25.5	26.9	21.9	25.7	22.6	28.6
>5	9.5	6.9	11.4	8.6	11.1	11.1
Education						
Illiterate	0.3	-	-	-	0.4	0.3
Elementary school graduate	0.1	-	0.5	0.4		0.3
High school graduate	5.8	4.8	4.1	6.9	6.4	6.4
Technical/vocational high school graduate	0.6	1.0	-	0.4	0.9	0.7
Postsecondary technical school	1.3	0.7	1.4	1.2	1.3	2.0
Graduate of institute of vocational education	2.4	1.0	2.3	2.4	2.1	4.0
University/college student	18.4	21.0	13.7	21.4	17.1	15.5
College/university graduate	70.9	71.4	78.1	67.2	71.8	70.7
Occupation						
University/college student	23.1	32.8	19.6	25.3	17.9	16.5
Public officer	26.8	19.7	28.8	24.8	28.6	34.0
Self-employed	15.3	15.5	15.5	14.5	17.9	14.5
Employee	18.6	15.9	17.8	19.3	21.4	18.5
House/Retired	2.5	2.1	2.3	2.4	3.8	2.4
Military service	0.5	1.0		0.6	0.4	0.3
Unemployed	8.0	6.9	12.8	7.7	6.8	6.7
Other	5.2	6.2	3.2	5.3	3.0	7.1
Monthly income						
None	6.5	5.5	6.4	6.3	7.3	7.1
<EUR 600	9.9	12.1	10.5	8.8	9.8	9.1
EUR 601–EUR 900	12.7	13.4	12.3	12.3	14.5	11.1
EUR 901–EUR 1200	15.5	12.1	16.9	17.1	15.8	14.8
EUR 1201–EUR 1500	13.0	13.8	13.7	12.8	13.2	11.8
EUR 1501–EUR 2200	20.2	21.7	15.5	19.8	19.7	23.2
EUR 2201–EUR 3000	11.4	11.7	13.2	10.6	10.3	12.1
EUR 3001–EUR 4500	6.1	4.5	6.8	7.9	4.3	5.7
EUR 4501–EUR 9000	2.3	3.1	2.3	2.0	1.3	2.7
>EUR 9001	2.4	2.1	2.3	2.0	3.8	2.4

**Table 4:** Allergies of the participants.

	Total	Innovators	Early Adopters	Early Majority	Later Majority	Laggards
Cases	1531	290	219	491	234	297
%	100	18.9	14.3	32.1	15.3	19.4
	Percentages %					
Allergy to fish/seafood products						
Yes	2.0	2.8	1.4	2.2	0.4	2.7
No	98.0	97.2	98.6	97.8	99.6	97.3
Allergy to insects						
Yes	7.6	3.4	6.4	9.2	9.8	8.4
No	92.4	96.6	93.6	90.8	90.2	91.6

**Table 5:** Diet and behavior habits of the participants.

Consuming of Food Products/Habits	Innovators	Early Adopters	Early Majority	Late Majority	Laggards
Cases	290	219	491	234	297
Meat products	**2.49** ± 1.00	2.68 ± 1.06	2.51 ± 1.00	2.62 ± 1.09	**2.76** ± 1.08
Dairy products	2.09 ± 1.13	**2.07** ± 1.18	2.13 ± 1.14	2.10 ± 1.13	**2.24** ± 1.21
Fish/seafood products	**3.13** ± 0.96	3.16 ± 0.86	3.20 ± 0.92	3.15 ± 0.90	**3.25** ± 0.92
Organic food products	3.39 ± 1.10	**3.21** ± 1.15	3.38 ± 1.03	3.26 ± 1.14	**3.44** ± 1.16
Body exercising	3.13 ± 1.17	**3.06** ± 1.16	3.24 ± 1.17	**3.29** ± 1.15	3.22 ± 1.25
Recycling	**2.00** ± 1.27	2.11 ± 1.36	2.19 ± 1.38	2.14 ± 1.28	**2.20** ± 1.45
Checking food labels	2.25 ± 1.12	**2.24** ± 1.21	**2.55** ± 1.26	2.25 ± 1.21	2.27 ± 1.22

Data are given as mean values ± SD; Notes: 1 = very often, 2 = often, 3 = sometimes, 4 = rarely, 5 = never; the lowest values are indicated in bold, while the highest values are both bold and highlighted (in grey) per question among the clusters.

**Table 6:** Categorical regression models or categorical regression coefficients and other statistics.

Model I: R^2^ = 0.174		Model II: R^2^ = 0.164	
Independent Variables	Relative Importance	Independent Variables	Relative Importance
**Municipality of residence**	**0.121**	**Consumption frequency of fish/seafood products**	**0.419**
**Age**	**0.113**	**Body exercising frequency**	**0.196**
**Number of children**	**0.147**	**Checking food label frequency**	**0.207**
**Occupation**	**0.347**		
**Monthly income**	**0.147**		
**Other**	**<0.100**	**Other**	**<0.100**
Number of household members	0.097	**Consumption** frequency of meat products	0.082
Education	0.017	**Consumption** frequency of dairy products	0.074
Area of origin	0.005	Recycling frequency	0.015
Gender	0.004	**Consumption** frequency of organic food products	0.007

Notes: with bold/highlighted the variables with highest relative importance.

**Table 7:** Reasons for adoption and rejection of food products containing insects in visible and non-visible form.

	Ranking Sequence ^1^	Mean: 1: Strongly Disagree, 2: Disagree, 3: Neutral, 4: Agree, 5: Strongly Agree	
**Reasons for adoption**		**Visible insect**	Non-visible insect *
Alternative protein source	(1, 1 *)	4.36 ± 0.78 ^a^	4.30 ± 0.68 ^a^
Curiosity	(2, 2 *)	4.14 ± 0.83 ^b^	3.95 ± 0.96 ^a^
High nutritional value	(3, 3 *)	3.99 ± 0.85 ^a^	3.92 ± 0.78 ^a^
Improve the living conditions of farmed animals (cattle, pigs, poultry)	(4, 4 *)	3.91 ± 0.98 ^a^	3.82 ± 0.98 ^a^
Environmental reasons/climate change	(7, 5 *)	3.72 ± 1.01 ^a^	3.80 ± 0.91 ^a^
Replace other products (e.g., beef)	(6, 6 *)	3.85 ± 1.03 ^b^	3.62 ± 1.06 ^a^
Reduce epidemic outbreaks possibility	(8, 6 *)	3.65 ± 1.10 ^a^	3.62 ± 0.94 ^a^
Seeking new sensations	(5, 7 *)	3.89 ± 1.07 ^b^	3.52 ± 1.15 ^a^
Nice taste/aroma	(9, 8 *)	2.97 ± 0.60 ^a^	2.95 ± 0.72 ^a^
Nutrition habitude	(10, 9 *)	1.99 ± 0.92 ^a^	2.28 ± 1.01 ^b^
**Reasons for rejection**		**Visible insect**	Non-visible insect *
Disgust	(1, 1 *)	4.90 ± 0.39 ^a^	4.83 ± 0.53 ^a^
Fear	(3, 2 *)	3.97 ± 1.17 ^a^	4.03 ± 1.21 ^a^
Not changing nutrition habitude	(4, 3 *)	3.94 ± 1.23 ^a^	3.93 ± 1.24 ^a^
Lack of trust for the production procedure	(5, 4 *)	3.88 ± 1.20 ^a^	3.74 ± 1.19 ^a^
Safety reasons	(6, 5 *)	3.85 ± 1.10 ^a^	3.71 ± 1.12 ^a^
Not have nutritional value	(7, 6 *)	3.79 ± 1.16 ^b^	3.41 ± 1.14 ^a^
Not know how to cook	(8, 7 *)	3.33 ± 1.38 ^a^	3.20 ± 1.25 ^a^
Appearance	(2, 8 *)	4.38 ± 0.93 ^b^	3.17 ± 1.25 ^a^
Not know how to find	(9, 9 *)	3.00 ± 1.40 ^a^	3.12 ± 1.29 ^a^
Vegan or vegetarian	(10, 10 *)	1.77 ± 1.23 ^a^	1.92 ± 1.33 ^a^

^1^ Numbers in parentheses indicate the ranking sequence according to the mean values; * values corresponding to non-visible insect; data are given as mean values ± SD; same-superscript letter indicates no significant difference (*p* < 0.05) between the responses about visible or non-visible insects.

## Data Availability

The original contributions presented in the study are included in the article, further inquiries can be directed to the corresponding author.

## Supplementary Materials

The following supporting information can be downloaded at: https://www.mdpi.com/article/10.3390/foods13193199/s1, Table S1: Translated information given to participants as “brief education” about the benefits of the practice of consuming insects as food.
