# Supplementary material for: How Do Greeks Feel about Eating Insects? A Study of Consumer Perceptions and Preferences
Source: Foods. 2024 Oct 8;13(19):3199. doi: 10.3390/foods13193199 (PMC11475659; doi:10.3390/foods13193199)
Supplement: Supplementary file 1 [file foods-13-03199-s001.zip › foods-3161574-supplementary.pdf]

foods

MDPI

How Do Greeks Feel about Eating Insects? A Study of Consumer Perceptions and Preferences

Alkmini-Anna Gkinali [1], Anthia Matsakidou [1,*], Anastasios Michailidis [2] and Adamantini Paraskevopoulou [1]

[1] Laboratory of Food Chemistry and Technology, School of Chemistry, Aristotle University of Thessaloniki, 54124 Thessaloniki, Greece; gkinalia@chem.auth.gr (A.-A.G.); adparask@chem.auth.gr (A.P.)
[2] School of Agriculture, Department of Agricultural Economics, Aristotle University of Thessaloniki,
54124 Thessaloniki, Greece; tassosm@auth.gr
* Correspondence: matsakidou@chem.auth.gr

Supplementary material

Table S1. Translated information given to participants as "brief education" about the benefits of the practice of consuming insects as food.

1. The World Health Organization highlights the urgent need to identify alternative protein sources, because of the global population increase. In the coming decades, ensuring adequate nutrition for everyone may become increasingly challenging. Edible insects, for instance, are not only rich in protein but also contain essential amino acids, iron, and vitamins, often surpassing the nutritional value of beef.
2. The environmental footprint of cattle farming and the cultivation of land for animal feed is significantly larger compared to that of insect farming. This includes higher CO2 emissions, greater water and energy usage, and increased reliance on fertilizers, pesticides, and the generation of both liquid and solid waste.
3. The average person unintentionally may consume up to 1 kg of insects annually. Over 2 billion people regularly consume around 2,000 species of edible insects, either as a nutrient source or a gourmet choice, due to their taste and versatility in cooking. Additionally, insect-derived foods like honey and carmine are commonly consumed.
4. The consumption of edible insects is as safe as consuming beef.
5. The virus that causes COVID-19 is extremely unlikely to be transmitted through the consumption of foods containing edible insects. Consuming edible insects instead of meat could help prevent the emergence of new pandemics. In many regions, there is a shortage of high-quality proteins due to the pandemic. The consumption of edible insects could be a viable solution to this food crisis
